# Effects of Spinal Cord Injury Site on Cardiac Autonomic Regulation: Insight from Analysis of Cardiovascular Beat by Beat Variability during Sleep and Orthostatic Challenge

**DOI:** 10.3390/jfmk7040112

**Published:** 2022-12-09

**Authors:** Pietro Guaraldi, Mara Malacarne, Giorgio Barletta, Giuseppe De Scisciolo, Massimo Pagani, Pietro Cortelli, Daniela Lucini

**Affiliations:** 1 IRCCS Istituto delle Scienze Neurologiche di Bologna, 40139 Bologna, Italy; 2 BIOMETRA Department, University of Milan, 20129 Milan, Italy; 3 Department of Biomedical and NeuroMotor Sciences (DiBiNeM), Alma Mater Studiorum–University of Bologna, 40123 Bologna, Italy; 4 Neurofisiopatologia, Azienda Ospedaliero-Universitaria Careggi, 50134 Firenze, Italy; 5 Exercise Medicine Unit, Istituto Auxologico Italiano, IRCCS, 20135 Milan, Italy

**Keywords:** autonomic nervous system, sleep stage, spectral analysis, sympathetic parasympathetic feedback, spinal cord injury site

## Abstract

Purpose: The goal of this study on Spinal Cord Injury (SCI) patients with cervical or thoracic lesion was to assess whether disturbances of ANS control, according to location, might differently affect vagal and sympatho-vagal markers during sleep and orthostatic challenge. We analyzed with linear and nonlinear techniques beat-by-beat RR and arterial pressure (and respiration) variability signals, extracted from a polysomnographic study and a rest–tilt test. We considered spontaneous or induced sympathetic excitation, as obtained shifting from non-REM to REM sleep or from rest to passive tilt. We obtained evidence of ANS cardiac (dys)regulation, of greater importance for gradually proximal location (i.e., cervical) SCI, compatible with a progressive loss of modulatory role of sympathetic afferents to the spinal cord. Furthermore, in accordance with the dual, vagal and sympathetic bidirectional innervation, the results suggest that vagally mediated negative feedback baroreflexes were substantially maintained in all cases. Conversely, the LF and HF balance (expressed specifically by normalized units) appeared to be negatively affected by SCI, particularly in the case of cervical lesion (group *p* = 0.006, interaction *p* = 0.011). Multivariate analysis of cardiovascular variability may be a convenient technique to assess autonomic responsiveness and alteration of functionality in patients with SCI addressing selectively vagal or sympathetic alterations and injury location. This contention requires confirmatory studies with a larger population.

## 1. Introduction

In the US the reported annual incidence of traumatic Spinal Cord Injury (SCI) is about 24–77 people per-million inhabitants, leading to a prevalence of about 1800 per million with an average age of 28.7–55 years at the time of trauma, mostly because of road accidents or falls [1]. People with an SCI have a poor quality of life because of major motor, sensory and autonomic dysfunction [2]. This latter aspect is receiving growing attention, considering in particular the recent improvement in our understanding of the underlying neural substratum [3] and the emerging possibility of electroceutical therapies to ameliorate attendant autonomic dysregulation [4] possibly through the utilization of ad hoc neuroprosthesis [5]. From the definition in 1921 of “the Autonomic nervous system” (ANS) as purely motor [6], to the description of the unbroken unity of the multitude of processes that govern the human organisms, through the continuous activity of the “paired antagonistic innervation” (vagal and sympathetic) of the internal organs [7], the autonomic nervous system is now viewed as a complex bidirectional, sensory–motor, afferent–efferent, input–output, positive–negative feedback cybernetic system [8]. Cardiovascular neural control is accordingly the result of continuous linear and non-linear [9] interactions between sympathetic (ergotrophic/excitatory) and vagal (trophotropic/inhibitory) sub-systems [10], whereby cranial and spinal nerves show analogous functionality [11]. Schematically, the periphery (inclusive of the intrinsic cardiac nervous system) [12] results are linked cranially to the Central Autonomic Network (CAN) [13] through multiple two-way neural channels. Of practical interest is the different pathway followed by parasympathetic (vagal) fibers that interconnect the medulla oblungata directly with the periphery [12] and the sympathetic fibers that start from the spinal cord [14]. A similar dual pathway is followed by afferents: either vagal (connecting with the nodose ganglion) and subserving negative feedback reflexes or sympathetic sensory fibers (connecting via dorsal roots to the spinal cord) subserving positive feedback reflexes [15]. Focusing on heart rate, the final neural pathway governs the SinoAtrial (SA) node activity through the combination of afferent and efferent vagal and sympathetic fibers [16] terminating in a final cardiac relay station [17]. In this context, vagal activation might play a beneficial role in the interplay between positive and negative feedback mechanisms as part of a long-term neural remodeling [18]. The anatomical and functional asymmetry of the entire circuitry [8] must therefore be accounted for when interpreting the effects of SCI [19]. Importantly, vagal circuits [16] may remain largely unaffected, while sympathetic circuits, embedded in the spinal cord, may lose or impair [20] their supraspinal control, hindering the management of relatively straightforward mechanisms, such as the negative feedback cardiac baroreflex [21]. A clinical experimental model particularly suited to study this two-way interaction could be the case of SCI, considering different (thoracic and cervical) [19] sites of lesion, a condition for which data regarding autonomic nervous system control are scarce [22]. In addition, autonomic functions at rest may appear normal, suggesting that cardiac autonomic regulation should be examined with autonomic challenges [23]. In this feasibility study we hypothesize that SCI, affecting the spinal site of the two-way autonomic neural circuitry [22], might impair sympathetic positive feedback reflexes [10], to an extent reflecting the length of spinal cord that is lost to central control [5]. Conversely, negative feedback reflexes would be selectively spared. These ones, in fact, depend on the vagal innervation, which is unaffected. Overall, we hypothesize that such a complexity may be better addressed using a system medicine approach [24]. In this way the homeostatic functioning of autonomic networks results from the interaction of negative and positive feedback mechanisms [10], producing a better control of a multitude of autonomic interactions. The mathematical complexity may be tamed using multivariate statistics, and advanced graphical tools [25] in the context of simplified neural networks [8]. Accordingly, we studied a small group of SCI patients, characterized by a thoracic or cervical lesion [19], and compared them to a group of sex and age matched control individuals. We considered excitatory challenges characterized by spontaneous and physiologically induced sympathetic activation [23] as produced by REM sleep [26] and passive orthostatism (tilt), respectively [27]. In order to study ANS, we employed autoregressive analysis of RR and systolic arterial pressure beat by beat variabilities, a technique which, in the last decades, has gained wide acceptance as a means to obtain proxies of autonomic regulation [27,28]. This non-invasive methodology furnishes several markers of autonomic control. In particular, frequency domain normalized indices (low and high frequency component of RR interval variability) reflect changes of sympatho-vagal balance [27], while time domain indices such as total variance (or total power) and indices derived from amplitude oscillations such as Alpha Index (proxy of baroreflex gain) [29], act as surrogates of vagal tone and reflexes [30]. Of potential importance is the observation that the depotentiation of sympathetic control (as assessed by direct measures of muscle sympathetic nerve activity, MSNA, in volunteers subjected to simulated microgravity) was characterized by reduced average MSNA and maintained oscillatory properties [31], once again indicating that absolute and normalized data can provide different, yet complementary information [32].

The goal of this small study was to appraise the feasibility to assess ANS regulation in SCI patients and to verify if the site of the spinal lesion might differently affect vagal and sympathetic markers derived from spectral analysis of HR and SAP variabilities both at rest and during autonomic challenges [33] keeping in mind that spinal cord circuits have a high level of local organizational reflex circuitry [14].

## 2. Materials and Methods

### 2.1. Study Population

After obtaining informed consent, 4 cervical SCI with tetraplegia (C4-C7, 4 males, age 41 ± 20) and 6 thoracic SCI with paraplegia (T2-T12, 6 males, age 37 ± 13) and 8 control subjects (CNT, 7 males, age 38 ± 10) underwent a 24 h video-polysomnography under bed-rest controlled conditions. Prior to data collection, all subjects were questioned regarding sleep-related breathing disorders (SRBD) symptoms and underwent a clinical examination to exclude pathological conditions that might affect the sleep–wake cycle. All patients and all controls were assessed for SRBD by means of a previous nocturnal cardio-respiratory monitoring.

### 2.2. Protocol

All subjects were studied during two experimental sessions.

Video-polysomnography session which consisted of 16 channels continuously recording for 24 h (Albert Grass Heritage^®^, Colleague TM PSG Model PSG16P-1, Astro-Med, Inc., West Warwick, RI, USA or Neurofax Electroencephalograph EEG-1200, Nihon- Kohden Corp., Tokyo, Japan), as per standard practice in the sleep laboratory of Bologna. We considered in the present study the electroencephalogram (EEG: C3-A2, C4-A1, O2-A1), right and left electro-oculogram, electromyogram of the mylohyoideus and left and right anterior tibialis muscles in order to define the different sleep phases and continuous ECG, Systolic Arterial Pressure (SBP) (Portapres^®^ Model-2, Finapres Medical Systems, Paasheuvelweg, Amsterdam, Netherland) and thoraco-abdominal breathing recording in order to perform an autoregressive spectral analysis of cardiovascular variabilities combined with non-linear assessments. A number of digital files of about 5 min duration were extracted manually for each participant. They corresponded to the following epochs: pre-hypnic rest, non-REM (NREM) sleep, REM sleep, and post-hypnic rest. REM sleep furnished a condition of spontaneously induced sympathetic activation [26].

A rest/tilt session, performed under audio and video-polygraphic monitoring (ANScovery Modular System, SparkBio Srl, Bologna, Italy) recording continuously: beat to beat BP waveform (Finometer Midi, Finapres Medical Systems, Amsterdam, The Netherlands), ECG, oronasal and abdominal breathing and peripheral vasomotor tone (Model 15LT, Grass Techonologies, Quincy, MA, USA) for 5 min while the subject was in the clinostatic position (rest) after 20 min of supine rest, and for 5 min in passive orthostatic position during head-up tilt at 65° (tilt) [34]. This latter condition may be considered as a physiologically induced sympathetic activation suitable for RR and systolic arterial pressure (SAP) variability analysis [27].

All parameters were acquired and sampled at a rate of 1000 Hz throughout the recording.

#### 2.2.1. Autonomic Analysis

Indirect indices of cardiovascular autonomic regulation were obtained from linear and non-linear computer analysis of the RR, systolic arterial pressure and respiration, as previously described [35,36] and currently in use in our clinic. This approach permitted sympatho–vagal interaction at the SA node (from spectral analysis of RR variability) to be estimated, as well as indices of the information domain (regularity index–RRRo- and three beat deterministic pattern-RRP_0v) [37]. We also analyzed bivariate indices from RR interval and blood pressure (and respiration) in order to compute a frequency domain index of the cardiac Baroreflex–alpha Index [29] and assessed the Low Frequency spectral power of SAP V, as a proxy of sympathetic vasomotor activity [38].

#### 2.2.2. Sleep Analysis

The sleep–wake cycle was visually scored in 30 s epochs according to the standardized criteria of Rechtschaffen and Kales [39], as light (stages N1 and N2) NREM sleep, deep (stage N3) NREM sleep and REM sleep.

For autoregressive spectral analysis we considered pre-hypnic wake (pre-hypnic rest), stage N3 (NREM sleep), REM sleep and post-hypnic wake (post-hypnic rest).

The study was approved by the local ethics committee (Istituto Scienze Neurologiche: number: 0069612, date: 8 July 2020). The study was performed in accordance with the ethical standards laid down in the 1964 Declaration of Helsinki and its later amendments. Informed consent was obtained from all individual participants included in the study.

### 2.3. Statistics

Anonymized data are presented as mean ± SD. Significance of observations was estimated using Linear Mixed Model considering groups, conditions and interaction. The level of significance was set at *p* < 0.05. We utilized instead a non-parametric test (Jonckheere Terpstra) to compare the paired changes between NREM (basal) and REM sleep and from rest to tilt. We also employed a Linearity Test and Spearman correlation following the classical rank transformation of Conover [40].

The small number of subjects required consideration keeping in mind the feasibility nature of the study. Although it is not a barrier in itself [41], it calls however for careful interpretation, avoiding generalizations and preferring methods (such as Cohen’s d) that are not relatively affected by the number of subjects and focus on the magnitude (small = 0.2; medium = 0.5; large = 0.8; very large = 1.3) not on the probability of an effect [42]. Computations were performed using a commercial SW (SPSS v 27, IBM, Armonk, NY, USA).

## 3. Results

**Figure 1:**
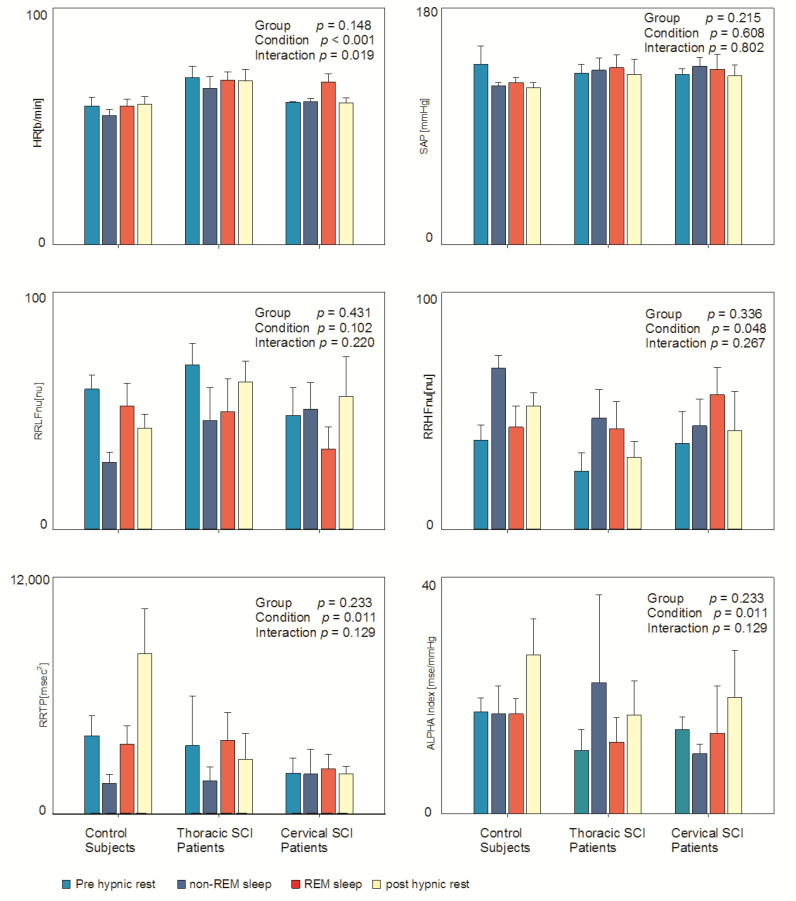
Hemodynamic and autonomic indices in the three examined groups (controls; thoracic and cervical Spinal Cord Injury) in different (spontaneous) conditions: pre-hypnic rest, NREM sleep, REM sleep and post-hypnic rest. Results of mixed model analysis are indicated in individual panels.

Table 1 depicts data derived from an autoregressive spectral analysis of RR and SAP variabilities in all the four considered conditions (pre-hypnic rest, NREM sleep, REM sleep, post-hypnic rest) in all three groups. The complexity of the protocol (four conditions) and the small number of subjects in the three groups limited the possibility to observe significant results. Nevertheless (see also Figure 1), it was possible to observe a clear reduction in Heart Rate (HR) during NREM sleep and its increase during REM sleep, particularly in control subjects (condition *p* = 0.001; interaction *p* = 0.019), while non-significant changes were observed in SAP. Moreover, it was possible to observe signs of the expected sympathetic activation (expressed by higher RRLFnu and reduced RRHFnu) during REM sleep in control subjects, while this finding was not present in SCI patients. Alpha index (marker of overall cardiac baroreflex sensitivity) appeared to be greater in all three groups in the morning (condition *p* = 0.011). RRTP (total power or variance of RR interval variability) showed significant changes during the different considered conditions (condition *p* = 0.011) in all groups. RRHFHz, which reflects respiratory frequency, was significantly different in the considered conditions (condition *p* = 0.003) being slowest during NREM sleep in all three groups.

**Figure 2:**
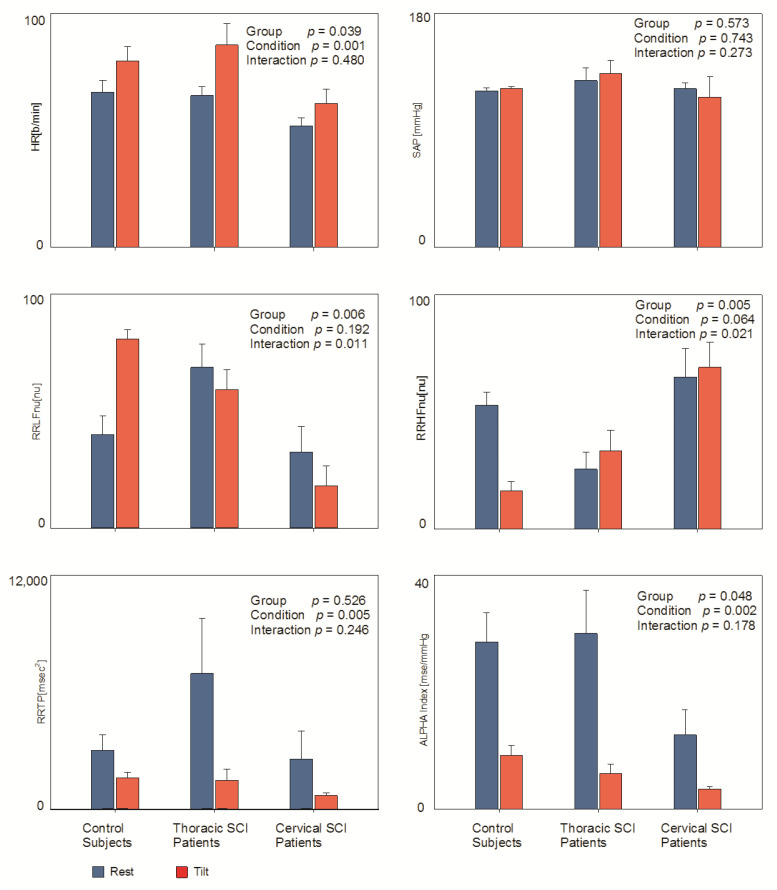
Hemodynamic and autonomic indices in the three examined groups (controls; thoracic and cervical Spinal Cord Injury) during rest and passive orthostatism (tilt). Results of mixed model analysis are indicated in individual panels.

Table 2 and Figure 2 report data derived from an autoregressive spectral analysis of RR and SAP variabilities during rest and tilt. This physiological sympathetic stimulus was clearly characterized by an increase in HR (condition *p* = 0.001) a reduction in RRTP (total power or variance of RR interval variability) (condition *p* = 0.005) and of the Alpha index (condition *p* = 0.002) in all three groups. With this stimulus, the Alpha index decreased significantly in all three groups, being smaller during rest in Cervical SCI patients (group *p* = 0.048, condition *p* = 0.002).

Of particular interest is the observation that, vice versa, RRLFnu (marker of prevalent sympathetic modulation to the sino atrial node) showed a significant different pattern of change in the three groups, increasing only in control subjects (group *p* = 0.006, interaction *p* = 0.011); specularly, RRHFnu (marker of prevalent vagal modulation to the sino atrial node) decreased only in controls (group *p* = 0.005, interaction *p* = 0.021).

**Figure 3:**
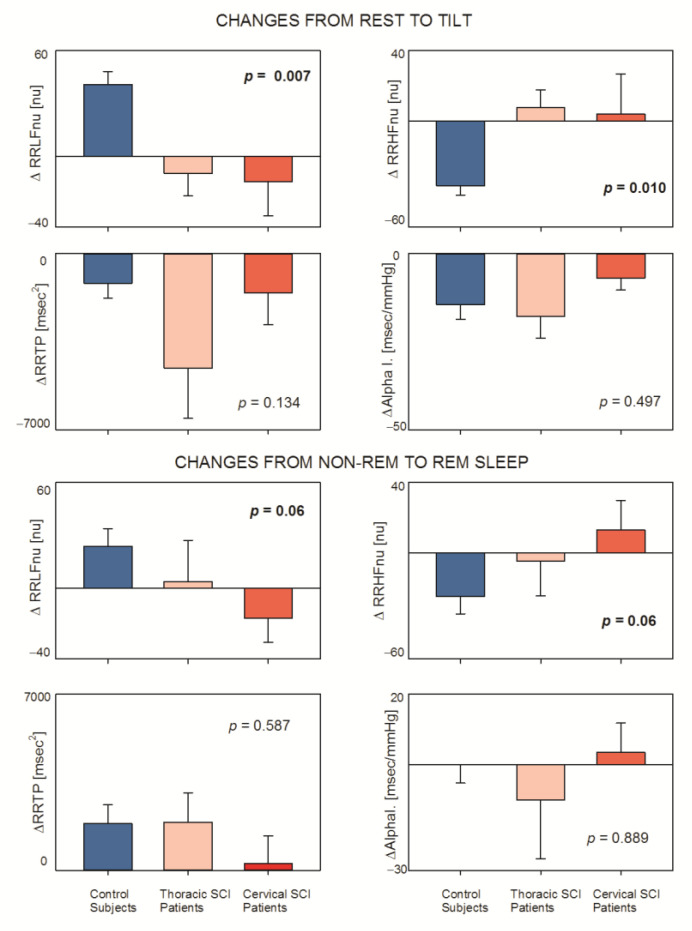
Effects of physiological (from rest to tilt) and spontaneous (from NREM to REM sleep) sympathetic challenges on selected autonomic indices in the three examined groups (controls; thoracic and cervical Spinal Cord Injury).

In order to clearly show the different responses with spontaneous (REM sleep) and physiological (tilt)-induced sympathetic activation stimuli, we performed a statistical analysis comparing the changes (Δ) from rest to tilt and from NREM to REM sleep considering markers of sympatho-vagal balance (RRLFnu and RRHFnu) and markers of vagal control (RRTP and Alpha index as a proxy of baroreflex control). Figure 3 clearly shows that in control subjects Δ RRLFnu (both rest–tilt and NREM sleep–REM sleep) was greater (*p* = 0.007 and *p* = 0.006, respectively) as compared to patients. The same responses were observed considering Δ RRHFnu (*p* = 0.010 and *p* = 0.006, respectively, Linearity Test *p* = 0.006 and *p* = 0.013). On the contrary, all three groups showed similar responses in ΔRRTP and ΔAlpha Index.

Table 3 reports the effect size of the described changes. It was expressed by Cohen’s d, a statistical index which is not affected by the number of subjects and rather focuses on the magnitude of the effect (48).

Notice that, considering the responses to tilt, a common powerful sympathetic stimulus, we observed an effect size of ΔRRLF and ΔRRHF clearly superior in controls, suggesting that SCI impairs sympathetic positive feedback reflexes, an impairment best evidenced during sympathetic stimuli. On the contrary, the effect size of ΔRRTP and ΔAlpha Index was similar in controls and patients, suggesting no significant interference of SCI with vagal mechanisms.

We also applied the Spearman correlation to the individual site of SCI (Table 4) against changes in autonomic indices induced by excitatory stimuli. This analysis permitted further observations regarding whether the location was significantly related to LF and HF changes (increase and decrease, respectively) only expressed in nu and with the strength of the stimulus (tilt). Notably, the correlation between SCI site, expressed as a metric of individual rank of extension of SC loss of functionality, and normalized spectral power of LF and HF nu, was very strong with intense stimuli, as with tilt. It became barely significant with the spontaneous shift to REM and was not significant at all with the shift from wakefulness to sleep.

Considering the variation induced by REM sleep, we observed a similar, yet reduced, pattern. Again, LF and HF changes were more strongly correlated using nu, although barely short of significance (*p* = 0.058, Table 4). Regarding non-linear autonomic indices, RRRo tended to increase in SCI patients (Group *p* = 0.035, Table 1) and decrease with NREM sleep (Condition *p* < 0.001). Likewise, RRP_0v increased in patients (Groups *p* = 0.042) and decreased with NREM sleep (condition *p* < 0.001). The reverse occurred with REM sleep, in keeping with the expected sympathetic shift. A specular effect was observed with RRP_2uv.

We also observed that among SAP V indices, LF SAP, a proxy of sympathetic vasomotor activity, increased only in controls during tilt (Table 3).

## 4. Discussion

In this study, we have reported preliminary observations on a small group of Spinal Cord-Injured patients with either proximal (cervical) or distal (thoracic) lesions. This particular clinical experimental model, in spite of the small number of subjects [41], showed that SCI impaired sympathetic positive feedback reflexes [15], while sparing selectively negative feedback reflexes that depend on an intact vagal innervation [43]. In particular SCI patients were characterized by reduced responses to spontaneous and physiologically induced sympathetic activation (as produced by REM sleep [26] and tilt, respectively [23,27] when considering RRLFnu and RRHFnu, markers of sympatho-vagal balance. On the contrary, similar responses were noticed when considering RRTP and Alpha Index, markers of vagal control [10]. Impaired responses were observed regarding LF SAP (a proxy of sympathetic vasomotor regulation) [10] during tilt in SCI patients. In this study, we chose two different excitatory challenges, characterized by spontaneous and physiologically induced sympathetic activation [23] as produced by REM sleep [26] and passive orthostatism (tilt), respectively [27]. Considering this model, the increases in RRLFnu (and conversely the decreases in RRHFnu) from rest to tilt and from NREM to REM sleep may be considered as markers of a shift in the sympatho-vagal balance towards sympathetic predominance [27]. It may be relevant to underline that in a previous paper [38] we showed that variations of RRLFnu were strongly correlated (*p* = 10^−6^) to analogous variations in the normalized (but not absolute) power of the low frequency components of spectral analysis of Muscle Sympathetic Nerve Activity variability. Moreover, the transient reduction in average MSNA produced by long term bed rest, was not accompanied by changes in spectral power distribution [31]. This profile may be considered analogous to what happens in the shift from the pre-hypnic state to non-REM sleep [44].

Considering stronger stimuli, in the present paper we observed that SCI patients presented strongly reduced responses, particularly evident with tilt, suggesting an impaired ANS functionality associated with SCI. These data are also supported by the Effects Size which was greater in controls than in patients, both for tilt and REM sleep. It may be worth recalling that the Effects size is not affected by the small number of studied subjects per se, and rather focuses on the magnitude of the effect [45]. The lesion site is an important determinant of the severity of clinical effect also regarding autonomic symptoms [19] with proximal (cervical) lesions being more serious. We observed that cervical SCI patients were characterized by smaller changes following the powerful sympathetic challenge (tilt) and underlined by a significant interaction (*p* = 0.011 for ΔRRLFnu and *p* = 0.021 ΔRRHFnu) and a significant linearity test (*p* = 0.006 for ΔRRLFnu and *p* = 0.013 ΔRRHFnu).

In spite of the small number of subjects, the individual location of SCI (Table 4) was strongly correlated to LF and HF changes only with the stronger stimulus produced by tilt and if expressed in nu. These differences were not present observing time domain indices such as RRTP and Alpha Index (derived from amplitude oscillations and proxy of cardiac baroreflex gain), which are surrogates of vagal tone and reflexes [30]. The same occurred considering the changes induced by tilt or by REM sleep, as expected considering that pathways followed by parasympathetic (vagal) fibers, that interconnect the medulla oblongata directly with the periphery, are not affected by SCI. Many studies in the literature have shown that time domain indices and indices of baroreflex gain reflect mainly parasympathetic control [30,43,46,47].

Taking into account the complexity of ANS control [8], we may not forget that the parasympathetic mechanisms such as cardiac baroreflex may be inhibited by the excitation of positive feedback reflexes [15].

In addition, the greater effect size of LF SAP in response to tilt observed in controls as compared to both SCI patient groups suggests that injury affects directly or indirectly the neural circuitry supporting positive feedback, sympathetic–excitatory responses, that rely on a functioning spinal cord (e.g., [48]). It should finally be recalled that the end result of autonomic regulation of the SA node is an integrated response to, both, sympathetic and parasympathetic antagonistic interaction, resulting, ideally, in a harmonious regulation [7]. In this context, SCI selectively impairs the ergotropic arm of the dual, bidirectional vegetative balance, and this impairment is progressively more evident with the more proximal location of injury [7,49], and with more powerful stimuli, such as tilt. It is also possible to approximately quantify the disturbance of autonomic regulation induced by SCI by imagining that the extent of damage is related to the (volumetric) residual cord length that is involved (by the number of metamers that are still working) [5].

Limitations of the study: This study presents some limitations that need to be considered. Firstly, the complexity of the protocol and the small number of subjects in the three groups limited the possibility to observe statistically significant results and their generalizability. For instance, in this paper we observed, counterintuitively, that NREM sleep was characterized by the smallest value of RRTP of all three groups. We also have to underline the unique aspects of this study which were based on two sections comprising long term polysomnographic and rest–tilt recordings, permitting an evaluation of physiological and spontaneous changes in autonomic drive both in patients and controls. Moreover, we employed a complex statistical methodology capable of accounting for the complexity of the protocol and the bias represented by small number of subjects. The study was simply observational, and our intent was to assess the feasibility of the general project. From this perspective, the results appear encouraging and permit the design of a future study with a longitudinal plan that could be more appreciated by readers and stakeholders.

## 5. Conclusions

Here, we have reported data from a small observational study about the feasibility of assessing the functional derangement accompanying proximal or distal SCI utilizing loop models [43] and a combination of clinical/statistical tools [25]. Monovariate or multivariate analysis from cardiovascular variability [10] provided data that were descriptors of the functional impairment, according to the site of the injury [49]. Distal lesions appeared less functionally damaging than proximal ones [19], which implied a lesser impairment of spinal autonomic circuitries [14]. The application of multiple descriptors (HRV, RRV, alpha indices) using a multidimension clinical model [25] should improve the understanding and facilitate the utilization of ANS markers in the clinical practice of SCI patients [50] eventually introducing neuroprosthetic interventions to vicariate the loss of autonomic regulation produced in individual patients by SCI [51].

In this preliminary, feasibility investigation, the small study population should be gauged together with Cohen’s d, that is not affected by the number of subjects and focuses on the magnitude not on the probability of an effect. Overall, the present results may provide the necessary information to plan more extensive future investigations, or they may be used to estimate important parameters that are needed to design the clinical study [52] which would be necessarily based on larger populations and require greater investments.

## Figures and Tables

**Table 1:** Summary descriptive data of the three study groups during the four conditions considered during polysomnographic recordings.

		Groups			Significance	
Indices in the Four Conditions	Control Subjects	Thoracic SCI Patients	Cervical SCI Patients	Between Groups	Between Conditions	Interaction
	**Mean**	**Mean**	**Mean**			
HR 1 (b/min)	58.62 ± 10.74	70.61 ± 10.73	60.14 ± 0.75	0.148	**0.000**	**0.019**
HR 2	54.50 ± 7.88	66.13 ± 11.38	60.44 ± 2.89			
HR 3	58.61 ± 8.35	69.54 ± 7.96	68.69 ± 6.88			
HR 4	59.46 ± 9.15	69.35 ± 10.47	62.45 ± 9.43			
RR 1 (msec)	1058.28 ± 221.46	865.23 ± 128.35	997.81 ± 181.13	0.202	**0.000**	**0.026**
RR 2	1123.81 ± 184.06	930.46 ± 168.06	994.44 ± 46.99			
RR 3	1044.32 ± 167.24	871.61 ± 97.06	880.03 ± 87.94			
RR 4	1035.66 ± 200.09	882.07 ± 140.89	1007.87 ± 78.29			
RRTP 1 (msec^2^)	3952 ± 2922	3461 ± 5577	2062 ± 1532	0.233	**0.011**	0.129
RRTP 2	1541 ± 1278	1666 ± 1617	2009 ± 2529			
RRTP 3	3533 ± 2582	3711 ± 3180	2290 ± 1474			
RRTP 4	8117 ± 6406	2763 ± 2913	2024 ± 754			
RRLFa 1 (msec^2^)	1049 ± 894	652 ± 557	850 ± 1479	0.193	**0.038**	0.191
RRLFa 2	247 ± 137	595 ± 515	293 ± 265			
RRLFa 3	768 ± 539	622 ± 665	91 ± 137			
RRLFa 4	891 ± 797	1118 ± 1398	427 ± 384			
RRHFa 1 (msec^2^)	741 ± 680	404 ± 607	230 ± 216	0.390	0.556	0.669
RRHFa 2	914 ± 1005	542 ± 587	268 ± 233			
RRHFa 3	796 ± 1168	473 ± 473	87 ± 78			
RRHFa 4	1411 ± 1758	570 ± 826	287 ± 279			
RRLFnu 1 (nu)	59.19 ± 17.10	69.45 ± 20.42	48.08 ± 23.22	0.431	0.102	0.220
RRLFnu 2	28.26 ± 12.18	45.82 ± 31.13	50.66 ± 22.49			
RRLFnu 3	52.14 ± 27.09	49.64 ± 30.69	33.71 ± 19.02			
RRLFnu 4	42.64 ± 16.92	62.12 ± 19.93	56.10 ± 33.52			
RRHFnu 1 (nu)	37.46 ± 18.22	24.41 ± 17.25	36.21 ± 26.63	0.336	**0.048**	0.267
RRHFnu 2	67.92 ± 15.42	46.89 ± 26.86	43.66 ± 22.52			
RRHFnu 3	43.13 ± 25.28	42.42 ± 25.92	56.81 ± 23.10			
RRHFnu 4	52.06 ± 16.04	30.32 ± 15.17	41.55 ± 33.05			
RRLF/HF 1 (.)	2.86 ± 3.54	6.44 ± 7.73	3.40 ± 4.70	0.317	0.114	0.758
RRLF/HF 2	0.48 ± 0.32	2 ± 2.41	1.95 ± 2.2			
RRLF/HF 3	3.86 ± 6.96	3.79 ± 6.24	0.94 ± 1.16			
RRLF/HF 4	1.02 ± 0.79	5.64 ± 9.29	8.83 ± 15.64			
RRHFHz 1 (Hz)	0.27 ± 0.02	0.27 ± 0.02	0.32 ± 0.02	0.077	**0.003**	0.940
RRHFHz 2	0.26 ± 0.03	0.25 ± 0.06	0.28 ± 0.03			
RRHFHz 3	0.27 ± 0.03	0.26 ± 0.07	0.29 ± 0.03			
RRHFHz 4	0.25 ± 0.03	0.24 ± 0.04	0.27 ± 0.06			
RRRo 1 [.]	0.32 ± 0.10	0.38 ± 0.08	0.35 ± 0.21	**0.035**	**0.000**	0.230
RRRo 2	0.18 ± 0.05	0.25 ± 0.08	0.36 ± 0.21			
RRRo 3	0.32 ± 0.13	0.48 ± 0.11	0.56 ± 0.08			
RRRo 4	0.36 ± 0.11	0.45 ± 0.12	0.41 ± 0.19			
RRP_0v 1 (.)	25.73 ± 13.32	33.30 ± 16.14	39.82 ±30.51	**0.042**	**0.000**	0.637
RRP_0v 2	9.52 ± 6.92	16.46 ± 6.93	29.53 ± 28.27			
RRP_0v 3	35.26 ± 15.83	47.06 ± 18.95	58.08 ± 11.99			
RRP_0v 4	29.33 ± 17.28	42.67 ± 17.73	36.08 ± 23.39			
RRP_2uv 1 (.)	20.03 ± 7.67	11.91 ± 3.35	15.08 ± 10.83	0.066	**0.000**	0.248
RRP_2uv 2	34.75 ± 11	18.73 ± 4.84	23.16 ± 16.39			
RRP_2uv 3	21.34 ± 13.29	8.06 ± 2.67	6.19 ± 2.24			
RRP_2uv 4	16.67 ± 10.32	7.56 ± 3.97	15.69 ± 10.75			
SAP 1 (mmHg)	124.16 ± 8.53	137.31 ± 31.34	130.61 ± 13.36	0.215	0.608	0.802
SAP 2	120.76 ± 6.58	132.68 ± 21.12	135.58 ± 13.46			
SAP 3	123.37 ± 10.72	134.51 ± 21.99	133.48 ± 22.97			
SAP 4	119.52 ± 10.55	129.55 ± 25.15	128.27 ± 16.74			
SAPTP 1 (mmHg^2^)	41.95 ± 22.97	45.51 ± 41.57	13.98 ± 4.66	0.297	0.437	0.055
SAPTP 2	15.8 ± 9.41	49.31 ± 32.56	31.45 ± 17.47			
SAPTP 3	38.21 ± 20.59	101.97 ± 127.03	33.69 ± 44.51			
SAPTP 4	29.12 ± 14.26	35.78 ± 29.54	67.91 ± 101.71			
SAPLFa 1 (mmHg^2^)	5.15 ± 5.73	27.68 ± 35.39	4.15 ± 5.01	**0.004**	**0.036**	0.093
SAPLFa 2	4.85 ± 4.66	17.45 ± 20.58	1.42 ± 1.08			
SAPLFa 3	4.32 ± 4.05	3.14 ± 2.50	0.54 ± 0.76			
SAPLFa 4	7.09 ± 13.95	11.38 ± 17.21	2.22 ± 3.28			
Alpha Index 1 (msec/mmHg)	17.23 ± 6.61	10.66 ± 8.04	14.19 ± 4.23	0.604	0.292	0.784
Alpha Index 2	16.89 ± 13.31	22.15 ± 33.05	10.16 ± 3.26			
Alpha Index 3	16.85 ± 7.36	12.10 ± 9.31	13.59 ± 16.12			
Alpha Index 4	26.82 ± 17.25	16.72 ± 12.89	19.66 ± 15.92			
BRS 1 (msec/mmHg)	14.08 ± 5.61	11.36 ± 9.35	27.33 ± 18.77	0.342	**0.002**	**0.005**
BRS 2	16.79 ± 8.89	11.54 ± 10.44	5.72 ± 0.08			
BRS 3	13.25 ± 7.05	13.29 ± 9.97	9.64 ± 6.64			
BRS 4	24.09 ± 17.28	13.94 ± 11.75	11.03 ± 0.42			

Abbreviations: 1 = pre-hypnic rest (evening); 2 = NREM sleep; 3 = REM sleep; 4 = post-hypnic rest (morning); HR = Heart Rate; RR = interpulse interval; RRTP = total power from RR variability; RRLFa = absolute value of the spectral power of the Low Frequency component of RR variability; RRHFa = absolute value of the spectral power of the High Frequency component of RR variability; nu = normalized units; Hz = frequency in Hertz; RRRo = index of RR regularity; SAP = systolic arterial pressure; SAPTP total spectral power of SAP variability; SAPLFa = absolute value of the spectral power of the Low Frequency component of SAP.

**Table 2:** Summary descriptive data of the three study groups during Rest and Tilt conditions.

		Groups			Significance	
Indices in the Two Conditions	Control Subjects	Thoracic SCI Patients	Cervical SCI Patients	Between Groups	Between Conditions	Interaction
	**Mean**	**Mean**	**Mean**			
HR rest (b/min)	66.39 ± 12.35	64.95 ± 9.36	52.01 ± 5.71	**0.039**	**0.001**	0.480
HR tilt	79.79 ± 14.94	86.49 ± 22.79	61.63 ± 10.61			
RR rest (msec)	930.82 ± 177.53	938.66 ± 124.48	1162.77 ± 125.61	**0.020**	**0.000**	0.761
RR tilt	775.31 ± 149.96	727.92 ± 159.30	994.72 ± 185.71			
RRTP rest (msec^2^)	2527 ± 1583	5799 ± 5726	2152 ± 2047	0.526	**0.005**	0.246
RRTP tilt	1346 ± 561	1243 ± 1162	584 ± 175			
RRLFa rest (msec^2^)	438 ± 370	1612 ± 1148	143 ± 187	0.101	**0.003**	**0.002**
RRLFa tilt	450 ± 255	248 ± 341	26 ± 37			
RRHFa rest (msec^2^)	841 ± 821	983 ± 1430	200 ± 208	0.561	**0.011**	0.519
RRHFa tilt	86 ± 64	172 ± 317	51 ± 39			
RRLFnu rest (nu)	39.98 ± 19.87	68.78 ± 24.45	32.60 ± 18.88	**0.006**	0.192	**0.011**
RRLFnu tilt	80.85 ± 9.76	59.30 ± 21.00	18.17 ± 14.87			
RRHFnu rest (nu)	52.98 ± 13.23	25.56 ± 17.58	64.93 ± 21.01	**0.005**	0.064	**0.021**
RRHFnu tilt	16.32 ± 9.34	33.40 ± 21.75	69.09 ± 18.49			
RRLF/HF rest (.)	0.87 ± 0.54	4.43 ± 3.36	0.59 ± 0.41	0.098	0.085	**0.009**
RRLF/HF tilt	7.18 ± 5.58	3.02 ± 2.74	0.32 ± 0.35			
RRHFHz rest (Hz)	0.25 ± 0.05	0.26 ± 0.02	0.23 ± 0.02	0.246	0.662	0.497
RRHFHz tilt	0.26 ± 0.04	0.25 ± 0.04	0.25 ± 0.03			
RRRo rest (.)	0.30 ± 0.11	0.34 ± 0.11	0.38 ± 0.03	0.146	**0.000**	0.664
RRRo tilt	0.44 ± 0.08	0.54 ± 0.15	0.53 ± 0.03			
RRP_0v rest (.)	28.23 ± 15.10	30.35 ± 13.28	35.77 ± 3.33	0.573	**0.000**	0.635
RRP_0v tilt	48.02 ± 9.55	54.90 ± 9.55	51.59 ± 8.57			
RRP_2uv rest (.)	18.02 ± 13.90	13.15 ± 10.92	10.94 ± 1.17	0.623	**0.024**	0.489
RRP_2uv tilt	6.76 ± 3.77	5.42 ± 2.57	7.45 ± 1.93			
SAP rest (mmHg)	120.57 ± 5.30	128.48 ± 24.19	122.11 ± 7.57	0.573	0.743	0.273
SAP tilt	122.12 ± 3.66	133.80 ± 24.55	115.64 ± 27.64			
SAPTP rest (mmHg^2^)	17.89 ± 8.26	40.73 ± 26.54	25.80 ± 17.70	0.313	**0.006**	0.182
SAPTP tilt	31.70 ± 5.74	45.81 ± 33.09	60.81 ± 56.45			
SAPLFa rest (mmHg^2^)	3.22 ± 2.27	5.90 ± 9.06	1.48 ± 0.47	0.207	0.242	0.661
SAPLFa tilt	8.46 ± 3.68	6.74 ± 8.59	2.74 ± 2.60			
Alpha Index rest (msec/mmHg)	21.41 ± 9.22	22.52 ± 13.63	9.54 ± 5.56	**0.048**	**0.002**	0.178
Alpha Index tilt	6.94 ± 3.15	4.59 ± 2.85	2.60 ± 0.53			
BRS rest (msec/mmHg)	16.41 ± 6.76	17.01 ± 12.90	13.98 ± 7.24	0.474	**0.005**	0.657
BRS tilt	7.78 ± 3.06	4.56 ± 2.20	3.94 ± 1.08			

Abbreviations: HR = Heart Rate; RR = interpulse interval; RRTP = total power from RR variability; RRLFa = absolute value of the spectral power of the Low Frequency component of RR variability; RRHFa = absolute value of the spectral power of the High Frequency component of RR variability; nu= normalized units; Hz = frequency in Hertz; RRRo = index of RR regularity; SAP = systolic arterial pressure; SAPTP total spectral power of SAP variability; SAPLFa = absolute value of the spectral power of the Low Frequency component of SAP variability; Alpha Index = frequency domain index of cardiac baroreflex; BRS = time domain index of cardiac baroreflex.

**Table 3:** Effect size of the changes in selected spectral analysis indices from Rest to Tilt and from NREM to REM sleep in the three study groups.

		Groups	
	Control Subjects	Thoracic SCI Patients	Cervical SCI Patients
	Cohen’s d	Cohen’s d	Cohen’s d
Tilt-Rest			
ΔRRLFnu (nu)	**2.272**	−0.302	−0.432
ΔRRHFnu (nu)	**−2.893**	0.324	0.106
ΔRRTP (msec^2^)	**0.814**	**0.933**	0.720
ΔAlpha Index (msec/mmHg)	**1.398**	**1.186**	**1.154**
ΔLFsap (mmHg^2^)	**1.338**	0.058	0.579
REM Sleep–NREM sleep			
ΔRRLFnu (nu)	**0.854**	0.073	−0.626
ΔRRHFnu (nu)	**−0.907**	−0.101	0.392
ΔRRTP (msec^2^)	**−0.884**	−0.728	−0.118
ΔAlpha Index (msec/mmHg)	0.003	0.270	−0.205
ΔLFsap (mmHg^2^)	−0.088	−0.678	−0.708

Abbreviations: ΔRRLFnu = change in the spectral power of the Low Frequency component of RR variability expressed in normalized units (nu); ΔRRHFnu = change in the spectral power of the High Frequency component of RR variability expressed in normalized units (nu); ΔRRTP = change in the total power of RR variability; ΔLFsap = change in the LF component spectral power of SAP V. Cohen’s d uses the sample standard deviation of the mean difference. It is not affected by the number of subjects and rather focuses on the magnitude of an effect. A value ≥ 0.8 suggests a meaningfully large effect (48).

**Table 4:** Spearman correlation between changes in autonomic indices during spontaneous inhibitory (wake to sleep) or spontaneous excitatory (non-REM to REM sleep) or physiologically induced events (rest to tilt) and position of Spinal Cord Injury.

	Wake-sleep	NonREM-REM	Rest-tilt	
HR	−0.568 *	0.252	−0.089	Correlation index
	0.017	0.329	0.753	Significance
RR Mean	0.606 **	−0.122	−0.111	Correlation Index
	0.01	0.642	0.694	Significance
RR TP	−0.593 *	−0.351	−0.185	Correlation Index
	0.012	0.168	0.51	Significance
RR LFa	−0.308	−0.602 *	−0.262	Correlation Index
	0.229	0.011	0.345	Significance
RR HFa	−0.347	−0.279	0.34	Correlation Index
	0.173	0.277	0.215	Significance
RR LFnu	−0.106	**−0.467**	−0.709 **	Correlation Index
	0.685	0.059	0.003	Significance
RR HFnu	0.153	**0.468**	0.646 **	Correlation Index
	0.559	0.058	0.009	Significance
RR Ro	−0.218	0.212	0.294	Correlation Index
	0.4	0.414	0.288	Significance
SAP Mean	−0.07	−0.118	0.018	Correlation Index
	0.79	0.653	0.948	Significance
SAP TP	−0.497 *	−0.336	0.089	Correlation Index
	0.042	0.187	0.753	Significance
SAP LFa	−0.022	−0.201	−0.36	Correlation Index
	0.933	0.44	0.188	Significance
AlphaM	−0.171	−0.16	0.116	Correlation Index
	0.512	0.538	0.68	Significance
BRS	−0.145	0.26	−0.151	Correlation Index
	0.653	0.415	0.59	Significance
RRP_0v	−0.266	0.037	0.063	Correlation Index
	0.339	0.896	0.824	Significance

Abbreviations HR = Heart Rate; RR = interpulse interval; RRTP = total power from RR variability; RRLFa = absolute value of the spectral power of the Low Frequency component of RR variability; RRHFa = absolute value of the spectral power of the High Frequency component of RR variability; nu= normalized units; Hz = frequency in Hertz; RRRo = index of RR regularity; SAP = systolic arterial pressure; SAPTP total spectral power of SAP variability; SAPLFa = absolute value of the spectral power of the Low Frequency component of SAP variability; Alpha Index = frequency domain index of cardiac baroreflex; BRS = time domain index of cardiac baroreflex; RRP_0v = non variation three beats pattern. * *p* < 0.05; ** *p* < 0.01.

## Data Availability

Data will be available on justified request. We do not uploaded the data considering the paucity of patient numbers which may not guarantee privacy.

## References

[1] Hagen E.M., Rekand T., Gilhus N.E., Grønning M. (2012). Traumatic spinal cord injuries—Incidence, mechanisms and course. Tidsskr. Den Nor. Legeforening.

[2] Anjum A., Yazid M.D., Daud M.F., Idris J., Ng A.M.H., Naicker A.S., Ismail O.H.R., Kumar R.K.A., Lokanathan Y. (2020). Spinal Cord Injury: Pathophysiology, Multimolecular Interactions, and Underlying Recovery Mechanisms. Int. J. Mol. Sci..

[3] Salavatian S., Ardell S.M., Hammer M., Gibbons D., Armour J.A., Ardell J.L. (2019). Thoracic spinal cord neuromodulation obtunds dorsal root ganglion afferent neuronal transduction of the ischemic ventricle. Am. J. Physiol. Circ. Physiol..

[4] Craig A., Pozzato I., Arora M., Middleton J., Rodrigues D., McBain C., Tran Y., Davis G.M., Gopinath B., Kifley A. (2021). A neuro-cardiac self-regulation therapy to improve autonomic and neural function after SCI: A randomized controlled trial protocol. BMC Neurol..

[5] Squair J.W., Gautier M., Mahe L., Soriano J.E., Rowald A., Bichat A., Cho N., Anderson M.A., James N.D., Gandar J. (2021). Neuroprosthetic baroreflex controls haemodynamics after spinal cord injury. Nature.

[6] Langley J. (1921). The Autonomic Nervous System.

[7] Hess W.R. (1949). The Central Control of the Activity of Internal Organs. Nobel Lecture Physiology or Medicine 1942–1962.

[8] Fukuda K., Kanazawa H., Aizawa Y., Ardell J.L., Shivkumar K. (2015). Cardiac Innervation and Sudden Cardiac Death. Circ. Res..

[9] Levy M.N. (1988). Sympathetic-vagal interactions in the sinus and atrioventricular nodes. Prog. Clin. Biol. Res..

[10] Malliani A., Pagani M., Lombardi F., Cerutti S. (1991). Cardiovascular neural regulation explored in the frequency domain. Circulation.

[11] Saper C.B. (2002). The Central Autonomic Nervous System: Conscious Visceral Perception and Autonomic Pattern Generation. Annu. Rev. Neurosci..

[12] Manolis A.A., Manolis T.A., Apostolopoulos E.J., Apostolaki N.E., Melita H. (2020). The role of the autonomic nervous system in cardiac arrhythmias: The neuro-cardiac axis, more foe than friend?. Trends Cardiovasc. Med..

[13] Shouman K., Benarroch E., Chokroverty S., Cortelli P. (2021). Central Autonomic Network. Autonomic Nervous System and Sleep.

[14] Pagani M., Schwartz P.J., Banks R., Lombardi F., Malliani A. (1974). Reflex responses of sympathetic preganglionic neurones initiated by different cardiovascular receptors in spinal animals. Brain Res..

[15] Pagani M., Pizzinelli P., Bergamaschi M., Malliani A. (1982). A positive feedback sympathetic pressor reflex during stretch of the thoracic aorta in conscious dogs. Circ. Res..

[16] Schwartz P.J., Pagani M., Lombardi F., Malliani A., Brown A.M. (1973). A Cardiocardiac Sympathovagal Reflex in the Cat. Circ. Res..

[17] Armour J.A. (2008). Potential clinical relevance of the ‘little brain’ on the mammalian heart. Exp. Physiol..

[18] Wang S., Zhou X., Huang B., Wang Z., Zhou L., Chen M., Yu L., Jiang H. (2015). Spinal cord stimulation suppresses atrial fibrillation by inhibiting autonomic remodeling. Heart Rhythm..

[19] Rodrigues D., Tran Y., Guest R., Middleton J., Craig A. (2015). Influence of neurological lesion level on heart rate variability and fatigue in adults with spinal cord injury. Spinal Cord.

[20] Previnaire J.G., Soler J.M., Leclercq V., Denys P. (2011). Severity of autonomic dysfunction in patients with complete spinal cord injury. Clin. Auton. Res..

[21] Draghici A.E., Taylor J.A. (2020). Cardiovagal baroreflex gain relates to sensory loss after spinal cord injury. Aut. Neurosci..

[22] Silvani A., Calandra-Buonaura G., Dampney R.A.L., Cortelli P. (2016). Brain–heart interactions: Physiology and clinical implications. Philos. Trans. R. Soc. A Math. Phys. Eng. Sci..

[23] Sharif H., La Fountaine M.F., Wecht J.M., Ditor D.S. (2018). A call to reevaluate cardiac autonomic assessment after spinal cord injury. Am. J. Physiol. Circ. Physiol..

[24] Ahn A.C., Tewari M., Poon C.S., Phillips R.S. (2006). The limits of reductionism in medicine: Could systems biology offer an alternative?. PLoS Med..

[25] Lucini D., Solaro N., Pagani M. (2018). Autonomic differentiation map: A novel statistical tool for interpretation of Heart Rate Variability. Front Physiol..

[26] Somers V.K., Dyken M.E., Mark A.L., Abboud F.M. (1993). Sympathetic-Nerve Activity during Sleep in Normal Subjects. N. Engl. J. Med..

[27] Pagani M., Lombardi F., Guzzetti S., Rimoldi O., Furlan R., Pizzinelli P., Sandrone G., Malfatto G., Dell’Orto S., Piccaluga E. (1986). Power spectral analysis of heart rate and arterial pressure variabilities as a marker of sympatho-vagal interaction in man and conscious dog. Circ. Res..

[28] Malik M., John Camm A., Thomas Bigger J., Breithardt G., Cerutti S., Cohen R.J., Coumel P., Fallen E.L., Kennedy H.L., Kleiger R.E. (1996). Heart rate variability: Standards of measurement, physiological interpretation, and clinical use. Circulation.

[29] Lucini D., Pagani M., Mela G.S., Malliani A. (1994). Sympathetic restraint of baroreflex control of heart period in normotensive and hypertensive subjects. Clin. Sci..

[30] La Rovere M.T., Bigger J.T., Marcus F.I., Mortara A., Schwartz P.J. (1998). Baroreflex sensitivity and heart-rate variability in prediction of total cardiac mortality after myocardial infarction. Lancet.

[31] Ferretti G., Iellamo F., Pizzinelli P., A Kenfack M., Lador F., Lucini D., Porta A., Narkiewicz K., Pagani M. (2009). Prolonged head down bed rest-induced inactivity impairs tonic autonomic regulation while sparing oscillatory cardiovascular rhythms in healthy humans. J. Hypertens..

[32] Kerkhof P.L.M., Peace R.A., Handly N. (2019). Ratiology and a Complementary Class of Metrics for Cardiovascular Investigations. Physiology.

[33] Kyriakides A., Poulikakos D., Galata A., Konstantinou D., Panagiotopoulos E., Chroni E. (2017). The effect of level of injury and physical activity on heart rate variability following spinal cord injury. J. Spinal Cord Med..

[34] Corazza I., Barletta G., Guaraldi P., Cecere A., Calandra-Buonaura G., Altini E., Zannoli R., Cortelli P. (2014). A new integrated instrumental approach to autonomic nervous system assessment. Comput. Methods Programs Biomed..

[35] Badilini F., Pagani M., Porta A. (2005). Heartscope: A software tool addressing autonomic nervous system regulation. Comput. Cardiol..

[36] Toninelli G., Vigo C., Vaglio M., Porta A., Lucini D., Badilini F., Pagani M. (2012). DynaScope: A Software Tool for the Analysis of Heart Rate Variability During Exercise. Comput. Cardiol..

[37] Porta A., Guzzetti S., Montano N., Furlan R., Pagani M., Malliani A., Cerutti S. (2001). Entropy, entropy rate, and pattern classification as tools to typify complexity in short heart period variability series. IEEE Trans. Biomed. Eng..

[38] Pagani M., Montano N., Porta A., Malliani A., Abboud F.M., Birkett C., Somers V.K. (1997). Relationship Between Spectral Components of Cardiovascular Variabilities and Direct Measures of Muscle Sympathetic Nerve Activity in Humans. Circulation.

[39] Rechtschaffen A., Kales A. (1968). A Manual of Standardized Terminology, Techniques and Scoring System for Sleep Stages of Human Subjects.

[40] Conover W.J., Iman R.L. (1981). Rank transformations as a bridge between parametric and nonparametric statistics. Am. Stat..

[41] Pawelczyk J.A. (2006). Big concepts, small N. J. Physiol..

[42] Fritz C.O., Morris P.E., Richler J.J. (2012). Effect size estimates: Current use, calculations, and interpretation. J. Exp. Psychol. Gen..

[43] Kawada T., Yamamoto H., Hayama Y., Nishikawa T., Tanaka K., Sugimachi M. (2019). Contrasting open-loop dynamic characteristics of sympathetic and vagal systems during baroreflex-mediated heart rate control in rats. Am. J. Physiol. Integr. Comp. Physiol..

[44] Pagani M., Guaraldi P., Baschieri F., Lucini D., Cortelli P., Chokroverty S., Cortelli P. (2021). Interpreting heart rate variability in sleep: Why, when, and how?. Autonomc Nervous System and Sleep.

[45] Sullivan G.M., Feinn R. (2012). Using Effect Size—Or Why the *P* Value Is Not Enough. J. Grad. Med. Educ..

[46] Katona P.G., Jih F. (1975). Respiratory sinus arrhythmia: Noninvasive measure of parasympathetic cardiac control. J. Appl. Physiol..

[47] Kollai M., Koizumi K. (1979). Reciprocal and non-reciprocal action of the vagal and sympathetic nerves innervating the heart. J. Auton. Nerv. Syst..

[48] Bishop V., Lombardi F., Malliani A., Pagani M., Recordati G. (1976). Reflex sympathetic tachycardia during intravenous infusions in chronic spinal cats. Am. J. Physiol. Content.

[49] Wecht J.M., Krassioukov A.V., Alexander M., Handrakis J.P., McKenna S.L., Kennelly M., Trbovich M., Biering-Sorensen F., Burns S., Elliott S.L. (2021). International Standards to document Autonomic Function following SCI (ISAFSCI). Top. Spinal Cord Inj. Rehabil..

[50] Malmqvist L., Biering-Sørensen T., Bartholdy K., A Krassioukov A., Welling K.-L., Svendsen J.H., A Kruse A., Hansen B. (2014). Assessment of autonomic function after acute spinal cord injury using heart rate variability analyses. Spinal Cord.

[51] Wecht J.M. (2021). Management of blood pressure disorders in individuals with spinal cord injury. Curr. Opin. Pharmacol..

[52] Chasan-Taber L. (2022). Writing Grant Proposals in Epidemiology, Preventive Medicine, and Biostatistics.

